# Exploring the future of the diagnostic process in primary care: The value of making contradictions explicit using change lab methodology with healthcare professionals and patients

**DOI:** 10.1186/s12913-025-13915-y

**Published:** 2025-12-29

**Authors:** Esmée W. P. Vaes, Esther de Groot, Siamack Sabrkhany, Ton Bruining, Jochen W. L. Cals, Dorien Zwart, Alma C. van de Pol

**Affiliations:** 1 Julius Center for Health Sciences and Primary Care, University Medical Center Utrecht, Utrecht, The Netherlands; 2 Department of Family Medicine, Care and Public Health Research Institute (CAPHRI), Maastricht University, Maastricht, The Netherlands; 3 Ton Bruining Onderwijsimpuls, Eindhoven, The Netherlands

**Keywords:** Diagnostic process, Innovation, Healthcare systems, Primary care, Change laboratory

## Abstract

**Background:**

Over the past years many innovations are being developed aiming to improve diagnostic processes in primary care. Limited evidence is available on how those actually involved in those processes, in short-hand end-users (patients, general practitioners, and medical specialists such as radiologists), view innovation of the diagnostic process in primary care. Perspectives from end-users are necessary to ensure the effectiveness of initiatives designed to improve the diagnostic process. Therefore, end-users explored directions and opportunities for the primary care diagnostic process, facilitated by change laboratory methodology in which tensions and differences of insight are used as source of inspiration and learning.

**Methods:**

Directions and opportunities were explored in two study groups comprising nine and ten participants respectively (consisting of patients, general practitioners, and medical specialists) that both had four change lab sessions in a four-month period. In the analysis, the Cultural-Historical Activity Theory was used as theoretical framework. This theory helps to reveal the operation of different healthcare systems in which participants find themselves and associated tensions and contradictions, creating new directions for thinking, learning, and acting from and with one another.

**Results:**

Together with the participants, we identified tensions and contradictions operating within and between different activity systems relevant for diagnostic processes in primary care, like tensions that could arise which arise when more and faster diagnostics are available in primary care or when collaborating parties have other interests and motivations in innovations. By recognizing these tensions and contradictions, participants have formulated innovation directions and opportunities for the diagnostic process in primary care. End-users perceived a need for better exchange of and/or access to test results done in hospital to general practitioners, and they identified certain artificial intelligence imaging techniques as promising to improve the diagnostic process for acute complaints at the point-of-care. Delving into these directions and opportunities for adjusting and advancing the diagnostic process, we formulated criteria to be considered for identifying fruitful innovation projects.

**Conclusions:**

By discussing tensions and contradictions between systems new considerations for successful innovation of the diagnostic process were identified and criteria were formulated which increase the likelihood of delivering promising innovation projects.

**Supplementary Information:**

The online version contains supplementary material available at 10.1186/s12913-025-13915-y.

## Background

In healthcare it is a common goal to deliver efficient care of high quality and to continue innovating patient care. Innovation is necessary to meet the many challenges that our healthcare system is facing worldwide now and in the future. Partly due to an ageing population, the number of people with one or more chronic conditions is increasing and with it a growing need for care [1], while healthcare workforce is seriously diminishing [2]. Especially access to primary care, as vital part of healthcare performance [3], is increasingly challenging, as the general demand for care increases [4]. To ensure continued access to primary care, a well-organised and efficient diagnostic process is crucial, as it also acts as an important link between effective primary and secondary care. Therefore, improving the diagnostic process through innovation can help to meet future care demands.

Innovation of the diagnostic process, including the development and marketing of new diagnostic tests of the past years, has resulted in a shift in the organisation of the diagnostic process in primary care, making it more complex [5]. Although innovation is often perceived as the development of new tests and tools [6], it encompasses more than that. Innovation in healthcare is described as the introduction of new tools, concepts, ideas, processes, and services, all aiming to improve patient care [5]. It evidently also includes organisational, social, and ethical considerations [7].

Innovation is often seen as something good [6]. Not all diagnostic innovations, however, have proven to be successful in achieving the desired outcomes [8, 9]. These experiences suggest looking at a system-level, considering relations and dependencies between stakeholders and their contexts, to ensure innovation meets the needs of daily practice healthcare professionals and patients, in short-hand the ‘end-users’ of the diagnostic process.

Limited evidence is available on what these end-users envision for innovation of the diagnostic process in primary care. More insight into the different perspectives of these end-users could provide new ideas and areas for innovation of the diagnostic process. Therefore, we brought together a group of end-users to learn from each other’s perspectives. Change laboratory is a method which can facilitate this learning from different disciplines and perspectives.

### Theoretical framework of change laboratory

**Fig. 1:**
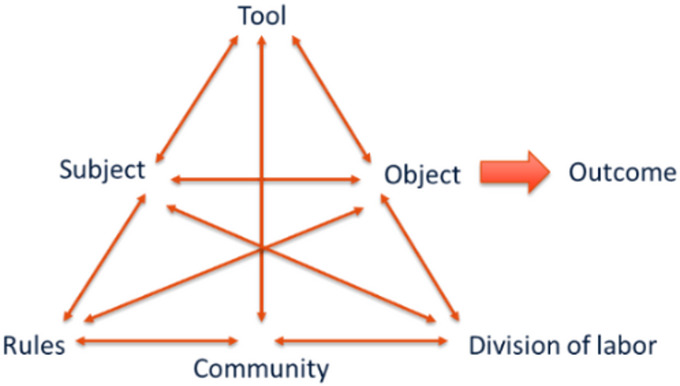
The basic structure of an activity system [10]

The change laboratory method is based on the Cultural Historical Activity Theory (CHAT), originating from previous work of Vygotsky and Leont’ev, and further developed by Engeström [10], and Virkkunen and Newnham [11]. According to the activity theory a person’s activity is organised in a system which consists of: tools he/she uses, objectives he/she pursues, rules that influence actions, communities in which the individual works and participates, and division of labour. This activity system illustrates complex interactions between an individual and its actions and the system in which he/she works involving social, technological, and organisational factors. Figure 1 shows a schematic representation of an activity system.

The change laboratory method involves an openness towards observing “tensions and contradictions” within and between activity systems and differences of insight between participants. Tensions and contradictions are used as source of inspiration and learning when exploring problems and solutions. Revealing contradicting viewpoints can help to cross both physical and sociocultural boundaries. These system level discussions offer potential for expansive learning, in which participants can create new practices (new working ‘models’) beyond the known and think of ways that are not yet there. This group learning is part of and takes place in a learning healthcare system. The change laboratory setting helps participants to collaboratively identify fruitful directions for innovations in a structured way.

### Study aim

The aim of this study was to explore directions and opportunities for innovation of the diagnostic process in primary care in a change laboratory setting, with the objective to involve end-users in system-level discussions about (im)possibilities, desires, and conditions for innovations of the diagnostic process and while doing so learn from other perspectives.

## Methods

### Design & setting

Between November ’23 and February ’24, we conducted in two similar study groups a series of each four change laboratory sessions (i.e. each group had four sessions) to explore directions and opportunities for innovation of the diagnostic process in primary care in two different regions of the Netherlands. In one study group all sessions were hosted live. In the other study group two out of four sessions were hosted online for practical reasons due to longer travel distances for participants. The sessions lasted between 60 and 90 min. The sessions were facilitated by a facilitator (TB) who had extensive experience with the change laboratory method. In each session at least two researcher-interventionists (EV, EdG, SS, AvdP) were present as observers who took notes during the sessions. The researcher-interventionists have a background as general practitioner (GP) or considerable knowledge of qualitative research methods. All sessions were audio-recorded and auto-transcribed by MS Teams.

### Organisation of the diagnostic process – the Dutch context

The change laboratory sessions took place in the region of Maastricht and in the region of Utrecht. These regions vary in population and in organisation of diagnostic facilities. This makes these regions suitable and interesting to include in our study, serving as examples for other regions that may share similarities with one of the two. Maastricht is located peripheral in the South of the Netherlands. The academic hospital provides secondary and tertiary care as well as primary care diagnostics in one geographical demarcated region. The vast majority of GPs order additional diagnostic tests, including medical imaging, laboratory tests, medical microbiology and pathology tests, through the diagnostic centre of this central academic hospital. This differs from the setting in the region of Utrecht, which is located west central and part of the Dutch main ring city. Multiple hospitals, including a large academic one, are located close to each other. Primary care laboratory diagnostics is provided by multiple organisations outside the hospitals.

### Participants

We purposely aimed to recruit healthcare professionals and non-healthcare professionals with an informed opinion on the topic of innovation of the diagnostic process. Participants had sufficient understanding of the Dutch language as the sessions were held in Dutch. People who might have a commercial interest were not included in this study. We asked all included participants and researchers to fill in a conflict of interest form before the start of the study to create transparency on possible interests and reasons for participation. Participants were recruited using a convenience sampling method via the network of the researchers in September and October 2023. We aimed to include approximately 10 participants in each study group, considering, based on the literature about change laboratories, this number appropriate for lively discussions with sufficient depth, and striving a balance in participating healthcare professionals working in primary and secondary care. Therefore, we included three GPs and three medical specialists working in a hospital in each study group. In one group, an internist participated who is working exclusively in primary care, advising many GPs in diagnosis and treatment. Moreover, two patients per study group were included to ensure they felt more comfortable sharing experiences with a group of professionals. After the first session in region 1, one participant dropped out because of a job change. Participant characteristics are available in Table 1.

**Table 1:** Participant characteristics

#	Gender, age range	Profession	Setting
***Region 1 Maastricht (n*** ***= 9)***			
1	Male, 30–39 years	General practitioner	Practice owner, specialized in emergency care
2	Male, 50–59 years	General practitioner-researcher	Primary care plus, specialized in musculoskeletal system and echography
3	Female, 40–49 years	General practitioner	Practice owner
4	Male, 40–49 years	Clinical chemist	Academic hospital
5	Female, 30–39 years	Medical microbiologist	Peripheral hospital
6	Male, 40–49 years	Radiologist	Academic hospital
7	Male, 40–49 years	Internist	Primary care
8	Male, 60–69 years	Patient	Not applicable
9	Female, 50–59 years	Patient	Not applicable
***Region 2 Utrecht (n*** ***= 10)***			
10	Male, 30–39 years	General practitioner	Practice owner
11	Male, 40–49 years	General practitioner	Locum GP
12	Female, 40–49 years	General practitioner	Practice owner
13	Female, 50–59 years	Clinical chemist	Peripheral hospital
14	Female, 40–49 years	Medical microbiologist	Peripheral hospital
15	Female, 30–39 years	Radiologist	Academic hospital
16	Female, 50–59 years	Relation manager for GPs	Peripheral hospital
17	Female, 30–39 years	Researcher in diagnostics	Primary care
18	Female, 10–19 years	Patient	Not applicable
19	Female, 40–49 years	Patient	Not applicable

### Data collection

At the beginning of each session the facilitator presented an agenda for that session. During the first session a short introduction into the Cultural Historical Activity Theory (CHAT) was given to participants. To help participants start the discussion on the topic and question each other on their perspectives and ideas, they received a fictitious patient trajectory on paper (first stimulus). The trajectories were based on narratives in literature and presented to GPs in our research team, after which the cases were altered based on their own professional experiences to make them recognisable and understandable for all participants. These patient trajectories were composed following a few predefined criteria (Table 2) which resulted from a group discussion in our research team including experienced GP-researchers. Each study group received a different patient trajectory, specifically designed to provide participants with a particular perspective, different from their own, as a starting point for discussion. Both trajectories are shown in Figures S1 and S2 (see Supplements).

**Table 2:** Criteria for developing the patient trajectories

1	The disease discussed in the trajectory must be frequently seen in primary care.
2	The symptom which is presented by the patient in the trajectory needs further diagnostic evaluation.
3	Establishing a diagnosis has a positive impact on the patient.
4	The diagnostic process must not be too simple.
5	The diagnostic process must not be too complex.
6	The trajectory must stimulate a discussion about innovation at multiple levels of the diagnostic process.

**Fig. 2:**
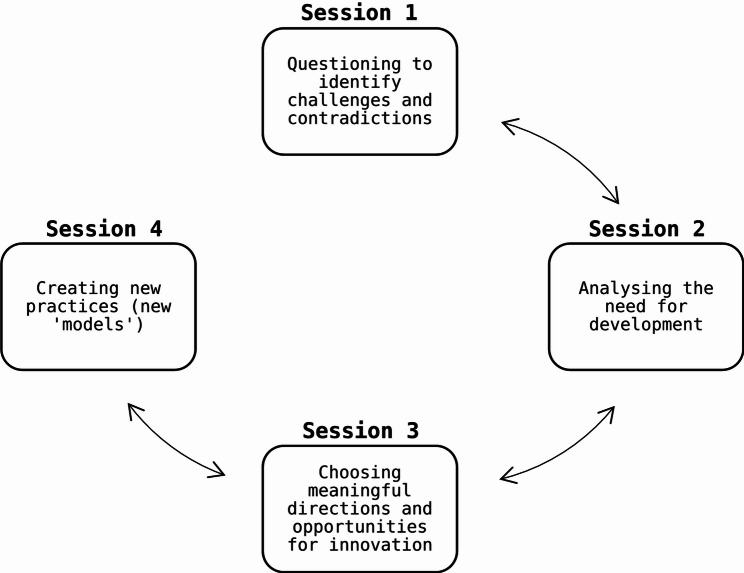
Overview of change laboratory sessions

Participants were asked to study the trajectory shortly and question each other on this (e.g. what do they think of the organisation of the diagnostic process in this case? Do they experience the same issues in their daily practice? Why do they experience these issues?). These questions were intended to elicit problems they experience in the diagnostic process in their own work practice and discuss contradictions surrounding these problems. By critically questioning each other about their experiences they were able to analyse the current as well as the historical origin of these problems. The discussions of the following sessions built further on this. Participants were encouraged to take initiative in the sessions during which they explored directions and opportunities for innovation of the diagnostic process. The intended goal for participants was to think of new ways (creating new ‘models’) of organising the diagnostic process. Researchers did not predefine how these outcomes should look like. An overview of the change laboratory sessions is visible in Fig. 2.

In between the sessions, participants received a summary of the previous session from the researcher-interventionists in writing, which served as preparatory material for the next session. These summaries contained conceptual tools (activity systems) which made contradictions more visual and was input for the next session. Participants were also asked to collect relevant materials (e.g. news articles, scientific research, etc.) which linked to the discussion during the sessions. Participants were stimulated to discuss the summaries with their colleagues and report new input by colleagues back at the next session.

### Analysis

The main goal of this study was to identify meaningful directions and opportunities for innovation of the diagnostic process. The researcher-interventionists (EV, EdG, SS, AvdP) and the facilitator (TB) came together in between the change laboratory sessions to interpret the discussions of the study groups. Based on these debriefings, the session recordings, and the fieldnotes made during the sessions, a summary of each session was made along with an outline for the next session.

After completion of all sessions two longer analysis sessions were organised during which also an external expert in the field of change laboratory (see acknowledgements) was invited to participate and reflect on the summaries of all change laboratory sessions. By discussing the input from the study groups and by analysing contradictions which participants identified researcher-interventionists distilled directions and opportunities for innovation of the diagnostic process in the form of criteria that participants believed should be followed to be able to innovate successfully. Because most relevant contradictions, according to participants, were discussed during the last change laboratory session, these contradictions were taken as starting point during the analysis and writing process.

### Reflexivity

The research team’s positionality played an important role in shaping both group dynamics and the analytic process. The GP-researchers brought intimate knowledge of general practice and could readily recognise the practical and emotional resonances of participants’ accounts. At the same time, their professional proximity required conscious attention to avoid assumptions of shared understanding. The healthcare researchers and the facilitator with expertise in change laboratory methods contributed an external perspective, often questioning taken-for-granted aspects of general practice. By making these positional influences explicit and discussing them throughout data collection, summary writing, and theme development, we were able to identify how our backgrounds shaped our interpretations. This reflexive engagement enhanced transparency and supported a more nuanced and balanced analysis.

### Ethics approval

This study did not require approval from an accredited medical ethics committee in the Netherlands as it did not fall under the scope of the Dutch Medical Research Involving Human Subjects Act (WMO). To ensure compliance with legislation and regulations, an independent quality check has been carried out in the UMC Utrecht (confirmation quality check 23U-0474). All participants provided written consent before participating in the sessions.

## Results

In the first change lab session, participants examined challenges in the diagnostic process of one patient trajectory (see Supplements). They commented on many of the issues that were implicitly anticipated by the research team, often relating them to their own professional practice. Then they explored directions and opportunities for innovation of the diagnostic process in primary care. In the next two sections, we highlight the directions and opportunities for innovation from both regions. Also, we describe observations of the research-interventionists during the course of the change laboratory sessions. Once all data was collected and analysed, the research-interventionists, using the knowledge gained in the first phase of analysis, developed criteria that can be considered when assessing an innovation project for fruitfulness. These criteria are described in the last section.

### Directions and opportunities for innovation of the diagnostic process

#### Results region 1

Different scenarios were discussed in which better, faster, and cheaper diagnostic possibilities closer to the patient could be provided in general practice. Participants thought of new tools and/or new settings for diagnostics. In one scenario smarter diagnostics were available in general practice. As example they discussed artificial intelligence (AI)-imaging techniques promising to improve the diagnostic process for certain acute complaints at point-of-care. In the activity system of GPs these new tools could be in potential timesaving as no referral to a diagnostic centre is needed and results are immediately available. In the activity system of patients these AI-imaging techniques at point-of-care allow faster results and diagnostics closer to the patient. On the contrary, participants acknowledged tensions which could occur when having more and faster diagnostics available for GPs. These diagnostic possibilities could also increase their workload if deployed more often (**contradiction A**). Participants thought that when having these quick tools available, the threshold to deploy them becomes lower (**contradiction B**). Moreover, more diagnostics could also result in more unexpected/secondary findings which could require additional diagnostics again (**contradiction C**), contributing to diagnostic uncertainty and increasing GPs workload.

Another scenario was transitioning more diagnostic facilities from hospital to primary care. These diagnostics could, for example, be delivered in bigger health centres where GPs and specialists both work as gatekeepers, and together triage which patients should be referred to secondary care, resulting in only more complex care in hospitals and low complex care in primary care. Participants discussed the tension between cheaper diagnostic possibilities in primary care and deploying more diagnostics if cheaper and readily available (**contradiction B**). On the other hand, more diagnostics ‘at the gate’ could eventually decrease hospital admissions (**contradiction D**).

By discussing these new practices for innovation of the diagnostic process, participants thought of new principles which are needed for innovation to become successful. Participants in region 1 had experienced in previous innovation projects that efficacy of an innovation was evaluated too early by grant providers and that reimbursements were no longer granted if the innovation had proved inefficient (**contradiction E**). Participants desired less focus on short-term results and more loosening of financial incentives, as they believed that innovations need more time to become part of a working process and to improve efficacy.

#### Results region 2

Whereas participants of region 1 thought of new tools and settings for innovating the diagnostic process, participants of region 2 thought of new ways to improve insight into and exchange of patient data between healthcare professionals. In this region multiple hospitals and primary care laboratory facilities are responsible for primary care diagnostics. For some specific patient groups, especially patients with a complex history, patients with (sub)acute complaints, and passers-by (no regular patients), GPs in this region often missed knowledge on relevant diagnostic information for these patients’ histories which could be relevant for diagnostic considerations and decisions at that moment. Therefore, GPs identified the wish to have insight into patient data from hospitals to be able to make better and faster diagnostic decisions. Next to a shared and up-to-date medication overview and patient’s history list between primary and secondary care, they wished access to all results of previous diagnostics (results of diagnostics, conclusion of the results, and interpretation of the conclusion by the specialist). To achieve these desires, participants perceived a need for better exchange of and/or access to history and test results done in hospital to GPs. Better data insight and/or exchange between primary and secondary care could also prevent unnecessary repeated testing, although the perception of how big this problem is differed. The group shared the perception that the current lack of easy data insight/access is not patient friendly.

At first, participants came up themselves with practical solutions for this problem. They posed, amongst others, AI as a possible tool that could help retrieve a summary of patient data in easy language understandable for GPs. The research-interventionists observed that the study group jumped quickly to discussing this practical solution, and enquired about the groups view of the underlying problem. An exploration of contradictions in or between systems causing these problems followed. Participants discussed why previous attempts for better data sharing failed. They saw that suppliers of care software have a different object than healthcare professionals, as suppliers have a financial drive. For this reason, suppliers have their own agenda and do not consider the needs in favour of good patient care from the working field, while participants believed that often the technological possibilities already exist. Participants identified this contradiction in objects between the activity system of the suppliers and the activity system of healthcare professionals as reason why despite the technological possibilities data sharing is still often a challenge in which suppliers do not work together with healthcare professionals (**contradiction F**).

In one of the later change lab sessions, participants realized that it is not their job to solve this contradiction, but rather to clearly formulate wishes which they have. Moreover, they proposed another strategy to communicate with suppliers of care software; to hand over a list with wishes they have regarding the software they are using. If the supplier cannot fulfil these wishes, healthcare professionals will contact another software supplier. Participants wanted to take more charge in this process.

### Observations during the course of the change laboratory sessions

#### High hopes for new technologies

Interestingly, in both regions we observed that participants were inclined to have high hopes for new technologies when discussing innovation of the diagnostic process. Initially, participants quickly came up with ideas for new technologies that they saw as a solution for future-proof care and described best practices for development and implementation of innovation. After hearing each other’s viewpoints, however, they agreed that innovation comprises more than only new technologies. By discussing tensions and contradictions they encountered in daily care practice, participants were able to envision innovative approaches, such as new concepts, ideas, processes, and services, that are not yet there, without introducing a new “appealing” technological tool.

#### Giving context to different healthcare systems

As researcher-interventionists, we noticed that the need for innovation is context-dependent, as participants (end-users) in both regions translated problems experienced in their own daily care practice into directions and opportunities for innovation meaningful to their specific working contexts. GPs in region 2 with multiple hospitals within short distance of each other experienced difficulties with insight in patient records of these different hospitals. Therefore, participants in this region perceived a need for better availability of all diagnostic data to innovate the diagnostics process. Whereas in region 1, where most GPs rely on one diagnostic facility in the academic hospital, participants did not perceive a need for better data insight to innovate diagnostics. They proposed new ways of delivering better and faster diagnostics in primary care instead of in hospital.

#### Inclination towards action progressed

Change laboratory helped participants to view their own position within the systems. Important to consider since innovation is not simply the solution for a problem; with each innovation the system in which this innovation is implemented must change too [12]. As each innovation could influence the whole system in which it is implemented, this method enabled participants to become more and more aware of changes within and between (current and future) systems. Moreover, the change laboratory method appealed to the initiative of participants for action. At first participants experienced the group discussions as being about issues that did not keep them busy in their daily work because they did not consider themselves part of the systems they spoke about. Over time, this changed and during the follow-up sessions participants talked about their roles and tasks within these systems toward new practices for innovation of the diagnostic process. The researcher-interventionists observed these developments within the participants as emancipatory. As the change lab sessions progressed the participants became more involved in the process and felt more comfortable in taking initiative. Also, although to a limited extent, colleagues of the participants were addressed to share their experiences and thoughts. At the end of the series of change laboratory sessions, their perspectives on what they needed changed from ‘a need to solve problems and posing solutions’ to ‘a need to address their desires’ thereby identifying and considering systemic contradictions. In this learning process, participants began to discuss the importance of crossing boundaries between different perspectives and diverse systems. The sessions in region 2 helped cultivate a shared motivation among participants to take charge and express their perspectives and desires. They felt it was essential to formulate key issues that needed addressing. Meanwhile, participants from region 1 recognized the need for new leading principles to innovate the diagnostic process in primary care.

### How can innovation be successful?

After completion of all change laboratory sessions, the research-interventionists reflected on the tensions and contradictions which participants from both regions discussed and they identified certain conditions necessary for any type of innovation to become a success. The research-interventionists formulated eight criteria to identify a fruitful innovation project application (Table 3).

#### Criterium 1, 2 & 3

In exploring new practices for better and faster diagnostic possibilities in primary care, the participants in region 1 identified a few contradictions which could occur (**contradictions A-D**). As participants acknowledged that GP workload could potentially increase with the availability of AI-imaging techniques at point-of-care, they thought it is important to know which systems are involved in the project application and how these systems are organised. This knowledge is needed to properly estimate whether an innovation can lead to a desirable decrease in workload. Participants thought that a project application of a large interdisciplinary group is in potential more relevant, as this united group supports this innovation. Participants believed that the systems involved in the application should be made clearly visible. Moreover, from the perspective of the participants the aim of a project should fit the needs that the project is trying to address. Therefore, not only needs of these systems should be evaluated, but also whether the project is proportionate to these identified needs. The project size should clearly fit the research aim. The researcher-interventionists heard in the discussion on AI-imaging techniques that participants valued involvement of patients highly, as they foresee that this innovation cannot be successful if patients do not support it.

**Table 3:** Criteria for identifying a fruitful innovation project brought forward by end-users

Criterium		Definition	Questions^*^	Contradictions^**^
1	*System involvement & visibility*	Evaluate the visibility, organisation, and openness to innovation of the system(s) of stakeholders involved in the project application.	Which system(s) is (are) involved in a project application? Are these systems sufficiently visible? Is attention paid to the organisation of these systems around an innovation? Are they open to innovation?	A - D
2	*Community needs*	Evaluate whether the community’s needs are clearly identified and if the scope of the project is proportionate to addressing those needs.	Is it sufficiently clear what needs the community has? Is the scope of the project proportionate to those needs?	A - D
3	*Patient preferences*	Evaluate the way patient preferences and differences between patients are considered.	Have patient preferences been considered and has sufficient attention been paid to differences between patients?	A - D
4	*Critical reflection*	Evaluate whether there is a critical reflection on the timeframe in which results and benefits of the project can be expected.	Is there sufficient critical reflection on the period in which results and benefits can be expected?	E
5	*End-user involvement*	Evaluate the way end-users are involved in the project.	How are end-users involved in the project and is that sufficient?	F
6	*Role of external systems*	Evaluate whether the proposed innovation assigns roles to other systems of stakeholders that are not originally involved in the application.	Are other systems placed in a certain role in the proposed innovation that were not involved in the application?	F
7	*Boundary crossing*	Evaluate whether the project pays sufficient attention to crossing boundaries between different perspectives.	Does a project pay sufficient attention to crossing boundaries between different perspectives (e.g. between patients, GPs and medical specialists, between GPs and software suppliers)?	F
8	*Interdependencies*	Evaluate whether interdependencies within and between systems of stakeholders are explicitly mentioned in a project application.	Are interdependencies (in systems, between systems) explicitly mentioned in a project application (e.g. between patients, GPs and medical specialists, between GPs and software suppliers)?	A - F

*The questions can be used when compiling an innovation project or evaluating such project application for fruitfulness

**The contradictions that were discussed in the different regions have resulted in these specific criteria

#### Criterium 4

Participants in region 1 also believed it is important that a realistic time goal is presented in the project application. Often it can be estimated beforehand that the intended goals are not realistic to meet within a certain time with the stakes proposed. Participants discussed that efficacy of an innovation should not be evaluated in a too early stage as an innovation needs time to make a process efficient (**contradiction E**).

#### Criterium 5, 6 & 7

When a specific test or tool is being proposed as an adjustment or advancement of the diagnostic process, the perspective of end-users of such a tool is essential. Participants in region 2 have experienced that end-users are insufficiently involved in the development and improvement of new products by software suppliers. As a result, the products and services did not match end-users’ needs and were no solution for problems end-users have in their daily practice (**contradiction F**). They argued the need to confront suppliers with their wishes, instead of accepting the products and services what suppliers are offering without adequate consultation of its users. Therefore, the way end-users are involved in a project is important to consider. Innovation should not be developed for, but together with, end-users. Participants suggested involvement of end-users during all phases of a project and ways to use their perspectives during these phases. Next to the involvement of end-users and patients in projects, participants discussed the importance of discussing involvement, roles, and tasks of all other relevant stakeholder systems in a project. To include all relevant systems in a project, participants discussed the importance of boundary crossing. Knowing how different stakeholders think about and work in their activity systems towards a common goal is important as perspectives can differ.

#### Criterium 8

When the research-interventionists discussed contradictions within and between current systems as well as future systems (which are not yet there), they concluded that contradictions should not be ignored nor strived towards ‘quick fixed’, but instead should be discussed. Thinking and discussing these contradictions can contribute to the success of an innovation by making interdependencies within systems more transparent. If energy is then expended to address the interdependencies and consider policies to mitigate those, the lessons learned could contribute to the success of an innovation.

## Discussion

The aim of this study was to get insight in the end-users’ viewpoints on fruitful directions and opportunities for innovation for the diagnostic process in primary care. Our two study groups did not only identify new diagnostic tools that could help them in diagnosing patients faster without increasing workload for GPs, but they also discussed new principles in collaborations and negotiations with external parties (for example suppliers of care software). Identifying and discussing tensions and contradictions within and between systems helped to develop new directions for thinking, and to explore acting with and learning from one another. After analysis of these contradictions, the researcher-interventionists compiled a list of criteria which can be used to identify, compile and/or evaluate an innovation project for fruitfulness.

### Relations to previous research

To our knowledge, this is the first change laboratory study addressing innovation of the diagnostic process in primary care. We believe that for innovation to become successful, tensions and contradictions in existing practices should be made explicit. Our contribution lies in demonstrating a novel process for eliciting them for the diagnostic process in primary care. An important feature of change laboratory is the active involvement of end-users, ensuring that outcomes are co-developed with the field rather than imposed top-down. In addition, it provides participants with a stronger sense of agency in the change process. In this light, participants shared previous experiences in which they were not sufficiently involved during an innovation. A literature review by Göttgens & Oertelt-Prigione [13] described different ways for end-user involvement in health innovation studies. In only a small number of studies included in this review end-users were involved as key stakeholders in all phases of the designing process of an innovation, whereas in most included studies end-users were only partially involved in this process. As needs and expectations of end-users may differ, as seen in this study on a regional level, it makes even more important to involve end-users to be more in line what matters to them.

The participants’ high hopes for new technologies correspond with what Janssen [6] described as ‘innovation logic’. In this innovation logic, innovation is often equated to new technologies and seen as inherently positive and promising for future improvement. Moreover, innovation is viewed as a linear process from development to implementation. As a result of these high hopes and linear thinking new technologies are often not critically reviewed for value and relevance, increasing the likelihood of disappointment if not meeting up to its intended results and expectations. We believe that the criteria formulated in Table 3 help to critically review innovation projects for potential fruitfulness.

A systematic literature review by Haring et al. [14] provided an overview of tensions in innovation processes within healthcare systems. Some of the tensions described can be directly linked to the eight criteria identified by end-users in the current study. For instance, one study included in the systematic literature review described the importance of considering patient needs to increase the success of an innovation [15], which aligns with our third criterium on ‘Patient preferences’. Another study included the systematic literature review highlighted the role of stakeholder collaboration and the capacity and openness to innovation as key factors for successful innovation. This corresponds to our first criterium on ‘System involvement & visibility’. The importance of looking at a system level for innovation is also supported by literature on implementation [16].

The criteria in our study have similar domains compared with the well-established Nonadoption, Abandonment, Scale-up, Spread, and Sustainability (NASSS) framework [17], particularly on involving end-users in the innovation process and acknowledging the broader systems in which these users operate and interact. The NASSS framework serves as an analytical tool to elucidate the degree of success or failure of a technological innovation. A key distinction, however, is that our criteria are not confined to technological innovations, thereby extending beyond the scope of the NASSS framework. While the authors of the NASSS framework recommend its use primarily as a means of structuring discussions rather than as a prescriptive checklist, we argue that our criteria can similarly facilitate structured discussion and are not intended as purely descriptive. Although various methods are available to facilitate such structured discussions, we chose change laboratory as it aims at transforming work practices through expansive learning. The Soft Systems Methodology (SSM) is another method that encourages system-level thinking too [18]. However, SSM places greater emphasis on fostering consensus among stakeholders. Our approach distinguishes itself from other frameworks which are often designed or chosen top-down, while these criteria were emerged through discussions with end-users who developed a situated understanding of change processes. The criteria can support a more evidence-based prioritization of initiatives and projects. In addition, designers (e.g. researchers, organisations allocating grants) may use these principles as a framework to refine and strengthen their ideas by systematically evaluating them against these criteria.

The findings of this study help us to better understand what is relevant to people at the primary care work floor by approaching the diagnostic process from a broader perspective and identifying contradictions. Contradictions between activity systems “are seen as vital forces for change and development” [19]. As such, in a complex healthcare system, various reasons may play a role at the same moment in time. Discussing about those different reasons and the inherent contradictions between some of those reasons is considered a source of learning. An example in our results is contradiction D where more diagnostics ‘at the gate’ could eventually decrease hospital admissions but was shown to lead to much discussion because hospitals had less profit. And even while contradiction A (more and faster diagnostics could increase workload for GPs) and contradiction B (deploying more diagnostics if cheaper and readily available) in our study were known from the literature [20], thus far this has been mostly considered important in hospital settings with their advanced diagnostic tools [21]. In our study, we have shown that the same challenges arise when more advanced tools are in the hands of GPs and as such being transparent about disadvantages of quick tools. Also, participants described suppliers as having their own agenda (contradiction F), which they saw as impeding the use of existing technological solutions. Earlier research has highlighted regulatory restrictions on sharing personal health data as a critical barrier [22], yet our participants did not perceive regulation as the main explanation for persistent difficulties in data sharing. This nuance suggests that while our findings align with prior studies in acknowledging barriers to information exchange, they also diverge by emphasizing relational and organisational dynamics over regulatory aspects. Taken together, our study both acknowledges earlier evidence and introduces new perspectives by highlighting contradictions and relational factors between those aspects uncovered in that earlier evidence that are less prominent in current literature.

### Strengths and limitations

A strength of this study lays in its unique insight gained from discussions by end-users of diagnostics into their perceptions on innovation of the diagnostic process. Their view is indispensable to identify innovation directions workable for practice. Moreover, the facilitator (TB) was familiar with the Cultural Historical Activity Theory (CHAT) and experienced with facilitating change lab sessions. Therefore, he was able to stimulate and facilitate a discussion on a system level. For these system level discussions participants needed to cross boundaries to explore each other’s experiences and perspectives.

This study has some limitations. Due to the limited number of change lab sessions and limited time during these sessions, we chose a more pragmatic approach of using CHAT mainly after each session by the researcher-interventionists. Participants were therefore less involved in the visualization of the discussed activity systems. This limited preparation time and time investment during the sessions for participants. Therefore, we were able to find enough participants eager to participate in this study who could combine it with their own workload of daily practice. Our pragmatic approach thus made it possible to really involve daily practice professionals and patients (end-users) and engage in discussions with each other. Moreover, due to our research approach, which did not include an intervention and is built upon a qualitative design that aims to identify theoretical principles, our findings were not intended to be generalizable from a sample to a population, e.g. in the rest of the Netherlands or abroad. Instead, our findings—and particularly our change laboratory approach—may inform reflective discussions in other health systems and countries. Also, we propose our findings to be considered for adoption to other settings that are similar to ours (case-to-case translation [23]).

### Future research

Based on our findings, we are curious to evaluate the use of the eight criteria by project applicants (e.g. researchers) and organisations allocating grants and would encourage others to build upon the directions and opportunities that were identified. It would also be interesting to explore how these end-users, which was a loosely coupled group of participants working in different (healthcare) organisations, can be empowered to list their needs in terms of exchange of and/or access to history and test results and how they can deliver their desires to the involved suppliers, specifically how they could take charge. Finally, there may be other regions (in the Netherlands or internationally) where the organisation of the diagnostic process and involved systems differ from those described in this study, making it necessary to consider the involved systems in these regions into more detail.

## Conclusions

By recognizing tensions and contradictions operating within and between different systems, innovation directions and opportunities for the diagnostic process were identified by end-users of diagnostics. By exposing these tensions and contradictions end-users learned from each other. This resulted in new ways of thinking and new practices for the diagnostic process. Analyses of the contradictions brought up by end-users also led to a list of eight criteria to consider which increase the likelihood of delivering promising innovation projects.

## Supplementary Information

Below is the link to the electronic supplementary material.

Supplementary Material 1

## Data Availability

The data that support the findings of this study are available from the authors upon reasonable request and with the permission of UMC Utrecht.

## Abbreviations

AI: Artificial Intelligence
CHAT: Cultural Historical Activity Theory
WMO: Dutch Medical Research Involving Human Subjects Act
GP: General practitioner
NASSS: Nonadoption, Abandonment, Scale-up, Spread, and Sustainability
SSM: Soft Systems Methodology
