# Supplementary material for: Exploring the future of the diagnostic process in primary care: The value of making contradictions explicit using change lab methodology with healthcare professionals and patients
Source: BMC Health Serv Res. 2025 Dec 29;26:160. doi: 10.1186/s12913-025-13915-y (PMC12859913; doi:10.1186/s12913-025-13915-y)
Supplement: Supplementary file 1 — Supplementary Material 1 [file 12913_2025_13915_MOESM1_ESM.docx]

# **Supplements**


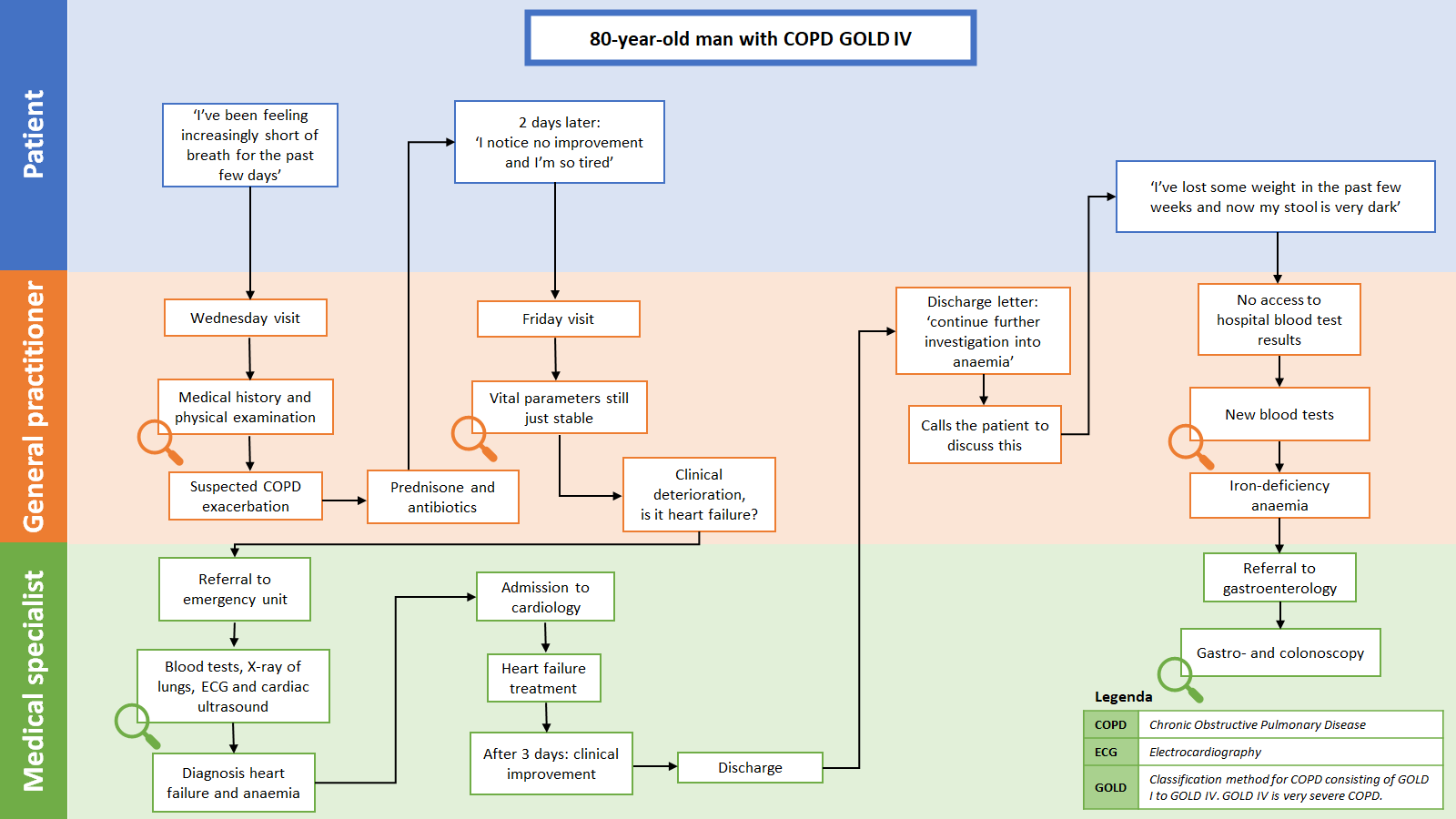


**Figure S1. Patient trajectory studied by participants in region 1 Maastricht (translated from Dutch)**


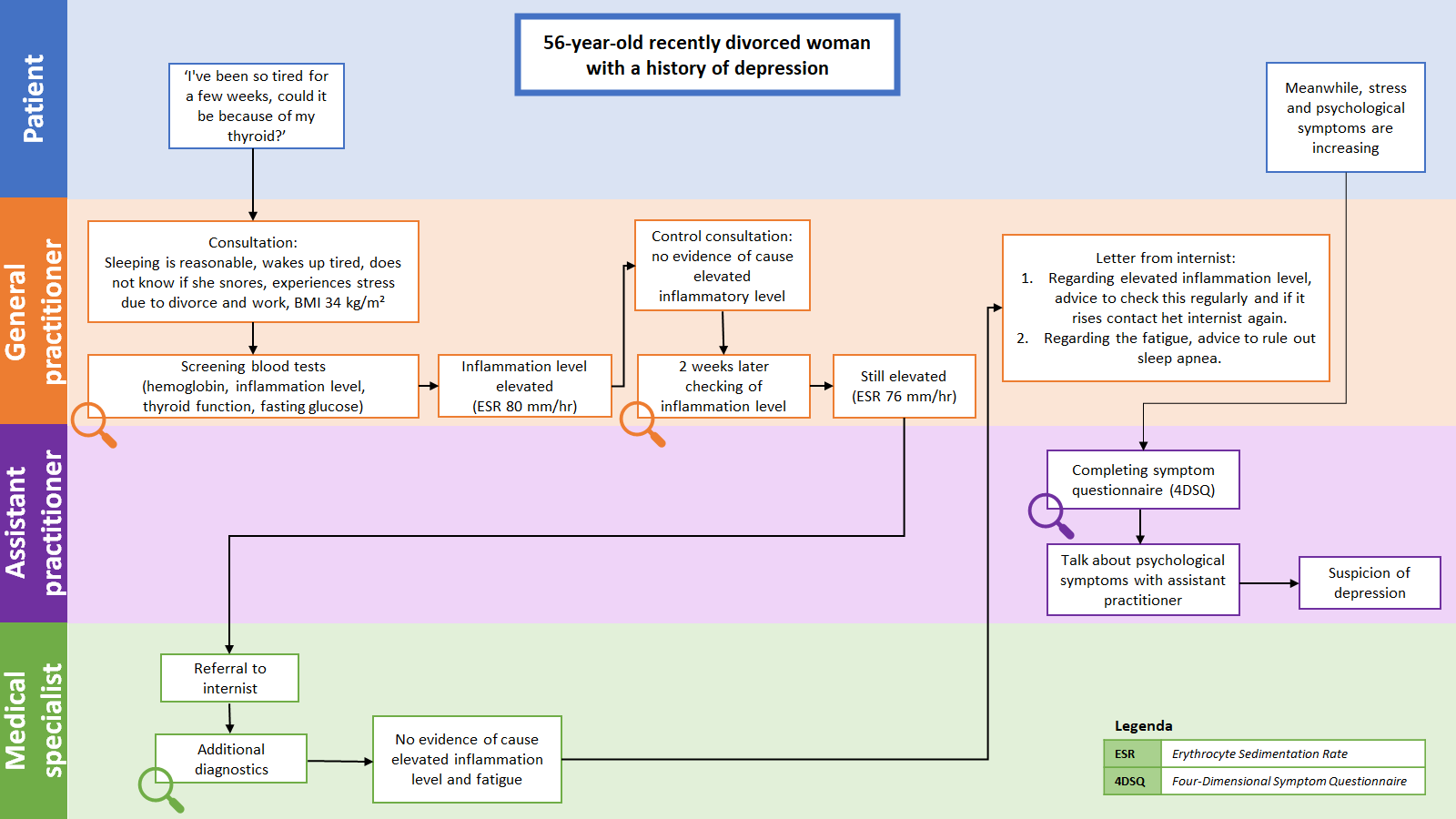


**Figure S2. Patient trajectory studied by participants in region 2 Utrecht (translated from Dutch)**
